# Large Anisotropy of Thermal Conductivity in Oriented Cellulose–Clay Composites

**DOI:** 10.1021/acsomega.5c00316

**Published:** 2025-06-16

**Authors:** Guantong Wang, Lengwan Li, Lilian Medina, Sivasankaran Harish, Jing Liu, Bin Xu, Rulei Guo, Cheng Shao, Takashi Kodama, Lars A. Berglund, Junichiro Shiomi

**Affiliations:** § Department of Mechanical Engineering, Graduate School of Engineering, 13143 The University of Tokyo, 7-3-1, Bunkyo-ku, Tokyo, 113-8656, Japan; † Department of Fiber and Polymer Technology, Wallenberg Wood Science Center, KTH Royal Institute of Technology, 10044 Stockholm, Sweden; & Institute of Engineering Innovation, Graduate School of Engineering, The University of Tokyo, 2-11, Yayoi, Bunkyo-ku, Tokyo, 113-0032, Japan

## Abstract

This study characterized the anisotropic thermal conductivity of clay/cellulose nanocomposites, an eco-friendly functional flame-retardant material exhibiting excellent mechanical properties, gas barrier properties, and biodegradability. Thermal conductivity anisotropy is important for flame-retardant materials. Low thermal conductivity in the through-thickness direction serves as a thermal barrier, whereas high thermal conductivity in the in-plane direction prevents local heat accumulation. We prepared a series of membranes of nanocomposites of montmorillonite clay platelets and cellulose nanofibrils via vacuum filtration/drying and measured their directional thermal conductivities as a function of the montmorillonite clay/cellulose nanofibril content. The results indicate that the through-thickness and in-plane thermal conductivities depend nonmonotonically on the clay content. The highest in-plane thermal conductivity reached 7.5 W m^–1^ K^–1^, exhibiting a maximum anisotropy of 30 for a clay content of 50%. Structural investigation via Raman spectroscopy revealed an enhanced planar alignment of the cellulose nanofibrils and indicated alignment of the clay platelets. The correlation between the degree of alignment and thermal conductivity anisotropy suggests that alignment increases the contact area between the cellulose nanofibrils and clay platelets, which enhances in-plane heat conduction by increasing the phonon transport path and impedes through-thickness heat conduction by enhancing phonon boundary scattering.

## Graphical abstract

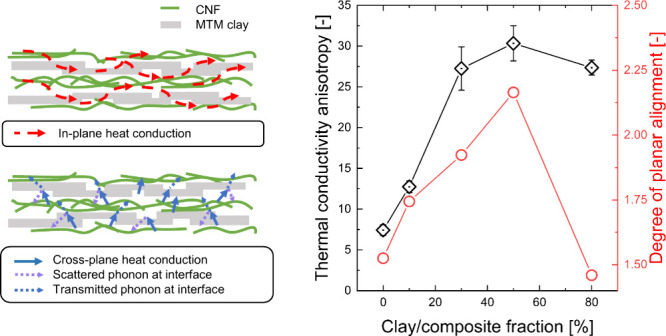

## Introduction

Flame-retardant materials, frequently used in transportation, the automotive industry, and protective garments for military applications,^1,2^ often rely on toxic halogens, such as bromine and chlorine.^3,4^ Recent studies on montmorillonite clay/cellulose nanofibril (MTM clay/CNF) composites as a green functional flame-retardant material^5–7^ indicate a promising solution owing to their excellent mechanical properties (Young’s Modulus up to 28 GPa), gas barrier properties, biodegradability, and ability to form foams of low density (40–120 kg/m^3^). Carosio et al. synthesized cellulose and montmorillonite clay foam via an ice-templating and sublimation processes and reported that light foams exhibited self-extinguishing properties and low combustion rates.^8^ When multilayered composites are subjected to fire, the montmorillonite phase forms protective surface barriers against oxygen on the outside and volatile substances on the inside. This ‘brick and mortar’ composite expands its structure during fire to form a char layer which prevents heat flow and insulates the underlying material core. Liu et al. showed that although pure CNFs exhibited high flammability and burned quickly and completely when subjected to fire,^9^ due to the introduction of montmorillonite nanoplatelets, the shape of the composite was preserved after a cone calorimetry test. In particular, at 50% montmorillonite content, the composite preserved its shape, and the flame was extinguished after removal of the heat source. Furthermore, such a dense CNF/MTM clay composite (50% MTM clay) exhibits superior heat shielding effect.^10^ In their experiment, when a flame torch was placed on one side of a 100 μm thick film for 1 min, increasing the surface temperature to 900 °C, the temperature on the back side was only 300 °C, resulting in a through-thickness temperature difference of more than 600 °C.

Recent works indicate the possibility that a polymeric nanocomposite component with a relatively high in-plane thermal conductivity may also contribute to heat shielding by enhancing heat conduction in the direction parallel to the surface (in-plane directions). Although CNFs are typically regarded as a thermal insulator,^11^ recent studies on CNF filaments have shown that their thermal conductivity can reach 14.5 W m^–1^ K^–1^, and thus, CNFs can be a high thermal conductor in the axial direction when highly aligned.^12^ Studies on CNF composites have also shown promotion of thermal conductivity. Song et al. synthesized a flexible film with CNF and graphene oxide via a vacuum-assisted self-assembly. A composite with high in-plane thermal conductivity (6.2 W m^–1^ K^–1^) was achieved^13^ via the correlated alignment of nano graphene oxide nanosheets. Tu et al. prepared a highly thermally conductive thin film by mixing CNFs with boron nitride. Interestingly, they found that with improved CNF alignment, the in-plane thermal conductivity of the composite increased nonmonotonically and achieved high thermal conductivity in the case of 50% boron nitride–50% CNF.^14^ Such an enhancement of anisotropy in thermal conductivity may explain the heat-shielding of montmorillonite clay/CNF flame-retardant materials^10,15^ by spreading heat radially in-plane, and thereby reducing the localization of heat. However, although the flame retardancy of the clay/CNF composite material has been confirmed through a horizontal flammability test in previous reports,^15–17^ their thermal conductivity has not yet been measured, particularly the anisotropic thermal conductivity.

In this study, we aim to explore the relationship between the anisotropic thermal conductivity of MTM clay/CNF composites. We prepare a series of membranes with layer-by-layer hierarchical structures using vacuum filtration and drying methods. Montmorillonite platelets acted as ‘brick’ with a thickness of 1.1 nm, and CNFs acted as ‘mortar’ with 3 nm in diameter and several micrometers in length.^15^ Directional thermal conductivity measurements were performed to characterize the dependence of the anisotropy ratio of the in-plane and cross-plane thermal conductivities on the montmorillonite clay/CNF fraction. In addition, Raman spectroscopy was performed to characterize the orientation of the CNFs and understand the mechanism of variation in thermal conductivity anisotropy.

## Experimental Section

During the preparation of montmorillonite (MTM) clay dispersion, a small amount of MTM powder was added to distilled water and sheared using a probe mixer for 30 min. Next, the mixture was ultrasonicated and centrifuged at 4500 rpm for 30 min. The well-dispersed upper clay suspension was then carefully extracted using a pipet and the remainder of the suspension was removed. During this process, sonication and centrifugation were repeated to 3–4 times until all the impurities within the suspension were removed.^15^ The density of the MTM clay suspension was approximately 2.86 g*·* cm^–3^ and composed of Cloisite-Na+ (BYK, Germany).

For the preparation of CNF dispersions, a CNF suspension consisting of approximately 13.8% hemicellulose and 0.7% lignin in the pulp fibril was prepared via mild enzymatic hydrolysis of sulfite pulp fibrils (Nordic Paper AB). Endoglucanase was added with mild heating. After the post treatment with the enzyme, the CNFs were washed and homogenized twice so that the final concentration of the suspension was approximately 1.4–2.0%.^15^

Next, nanocomposites were prepared. The aqueous suspension of CNFs was diluted to 0.05–0.15% using distilled water and then sheared for 10 min using a Super Turrax apparatus. The clay suspension was mixed with an aqueous suspension of CNFs for approximately 5 min. The clay-CNF suspension was then filtered under vacuum. The CNFs exhibit strong water retention properties, resulting in a relatively slow vacuum filtration process (>2 days) under a constant filtration pressure of approximately 0.1 MPa. A part of the test liquid was dried in an oven and weighed. The volume of slurry was calculated based on the weight ratio of the suspension before and after drying. Following filtration, samples were dried at 93 °C for 50 min to obtain the composite films. The content of clay in the nanocomposite films varied from 0 to 100%, and the thickness of each sample was around 80–110 μm. The details are summarized in Table 1.

**Table 1:** Density and Thickness of Nanocomposite Films

Sample	Density (g·cm^–3^)	Mean thickness (μm)	Cross-plane thermal conductivity (W m^–1^ K^–1^)	In-plane thermal conductivity (W m^–1^ K^–1^)
0%clay100%CNF	1.46 ± 0.15	96 ± 2	0.43 ± 0.04	3.18 ± 0.43
10%clay90%CNF	1.54 ± 0.2	83 ± 1.5	0.27 ± 0.03	3.38 ± 0.48
30%clay70%CNF	1.73 ± 0.18	88 ± 2.5	0.24 ± 0.03	6.63 ± 0.94
50%clay50%CNF	1.92 ± 0.15	89 ± 2	0.25 ± 0.02	7.44 ± 0.78
80%clay20%CNF	2.2 ± 0.2	81 ± 2.5	0.22 ± 0.02	6.04 ± 0.58
100%clay0%CNF	2.4 ± 0.15	101 ± 2	0.26 ± 0.02	2.2 ± 0.45

The overall flow of the vacuum filtration assembly is shown in Figure 1(a). The vacuum-filtration assembly helps form laminated nanostructures, as seen in the SEM and TEM images in Figure 1(b), where the clay particles are oriented in the in-plane directions of the composite, as in brick and mortar structures.^15,16^

**Figure 1:**
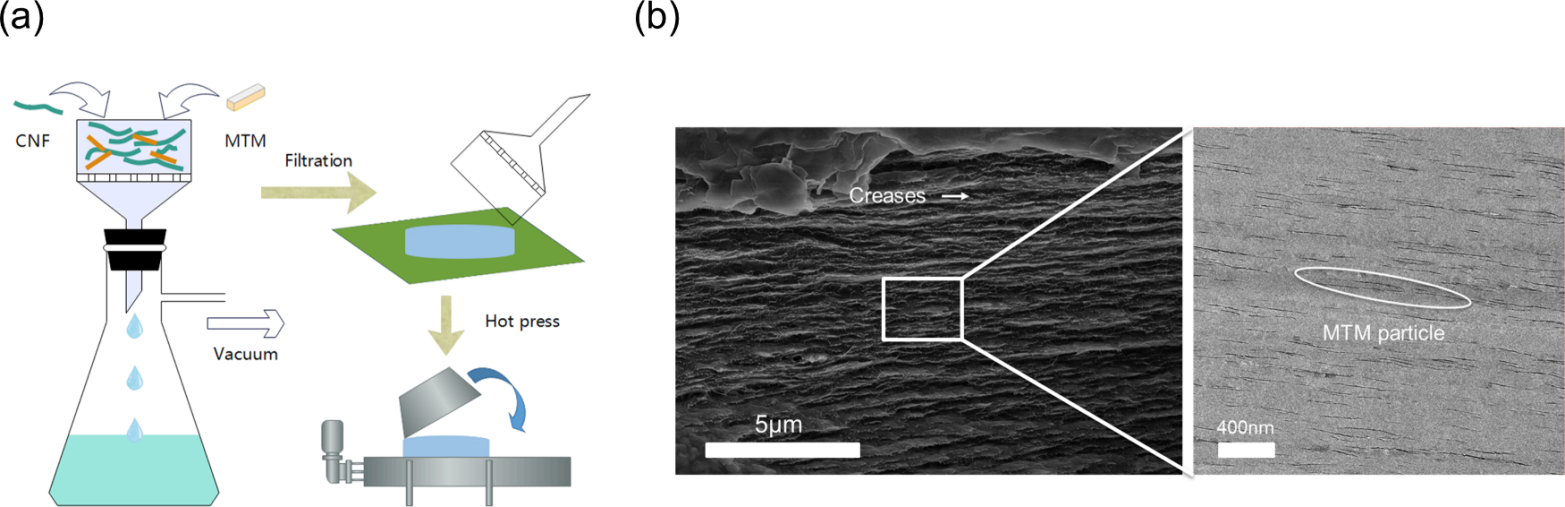
Preparation and characterization of MTM clay/CNF hybrid composites. (a) Schematic of the vacuum-filtration self-assembly method. (b) Scanning electron microscopy (SEM) and transmission electron microscopy (TEM) images of the composite cross-section, where the scale bars indicate 5 μm and 400 nm, respectively.

The cross- and in-plane thermal diffusivities were measured using the laser flash method (LFA 467 Hyper Flash, NETZSCH Company). An 8 mm square sample was prepared for the cross-plane thermal diffusivity measurement, and a rectangular sample of 25 mm in length and 8 mm in width was prepared for the in-plane thermal diffusivity measurement (owing to different modes for measuring thermal diffusivity). Because the clay/CNF sample was translucent, both surfaces of the sample were coated with gold of 100 nm in thickness to prevent detection of the temperature of the internal part. To prevent the reflection of the laser flash from the surfaces, graphite was coated on the top and bottom surfaces of the sample for better absorption of the incident light (a detailed schematic diagram is shown in Supporting Information Figure S-1).

During laser flash measurements, the temperature signal from the top detector was extracted, and the half time of the signal that reached the maximum value (*t*_50_) of the maximum temperature was used to calculate the thermal diffusivity α using eq 1:

1 $$\alpha =\frac{0.1388\times d^{2}}{t_{50}}$$

where *d* is the thickness of the measured sample. The measurements were performed three times to obtain the average value of thermal diffusivity

Temperature-dependent specific heat was measured using differential scanning calorimetry (DSC). First, two blank platinum pans were heated to 100 °C for benchmarking. Next, a reference sample (sapphire) and test sample were heated to 100 °C to evaporate the moisture inside. Then both pans were reheated again from 25 to 140 °C for the subsequent measurements. To maintain the reference and test samples at the same temperature during measurement, the difference in heat flux between the pans was monitored and used to finalize the heat capacity (details in Supporting Information Figure S-2).

We quantitatively investigated the structure of the CNFs in the composites using Raman spectroscopy (RENISHAW INVIA with a 633 nm laser, 1800 grating, 600 lens VIS, and CCD detector). Linearly polarized Raman spectroscopy for both incident and scattered beams was used to identify the CNFs’ orientations (details in Supporting Information Figure S-3).

The Raman shift peaks were strongly influenced by the orientation of the CNFs, which was evaluated by changing the polarization direction of the beams. Gierlinger et al. investigated the orientation of cellulose and lignocellulose microfibers using polarized Raman spectroscopy.^18^ In their analysis, latewood fibers were selected because CNFs in this type of wood fibers were confirmed to be aligned with the fiber axis at angles of less than 10°. The orientation of the CNFs was analyzed by changing the polarized light from perpendicular to parallel to the fiber axis. Based on the results, they found that most of the peaks changed with the polarization direction, except for the peak at 1377 cm^–1^, which represented the H–C–C, H–O–C, and H–C–O bonds (on the side of the molecule). Interestingly, the peak at 1096 cm^–1^ which corresponds to the C–O and C–C bonds along the cellulose molecular chain, changed significantly as they were sensitive to the alignment of the cellulose molecular chain. Therefore, in this study, linear polarization was utilized for both incident (parallel to the in-plane axial direction) and scattered light, and the degree of CNFs alignment was identified using the peak intensity at 1096 cm^–1^ normalized to the peak intensity at 1377 cm^–1^. Note that CNFs are expected to orient randomly in the plane, so what we mean here is the degree of alignment with respect to the plane, the “planar alignment.”

## Results and Discussion

The cross-plane and in-plane thermal conductivities of the composites (Figure 2a) with varying MTM clay contents are shown in Figure 2b and Figure 2c, where the thermal conductivity is calculated as

2 $$\kappa =\alpha \rho C_{p}$$

Here, κ is cross-plane or in-plane thermal conductivity, α is cross-plane or in-plane thermal diffusivity measured via the laser flash method, ρ is the density of the sample measured using Archimedes principle, and *C*_*p*_ is the heat capacity measured via DSC.

**Figure 2:**
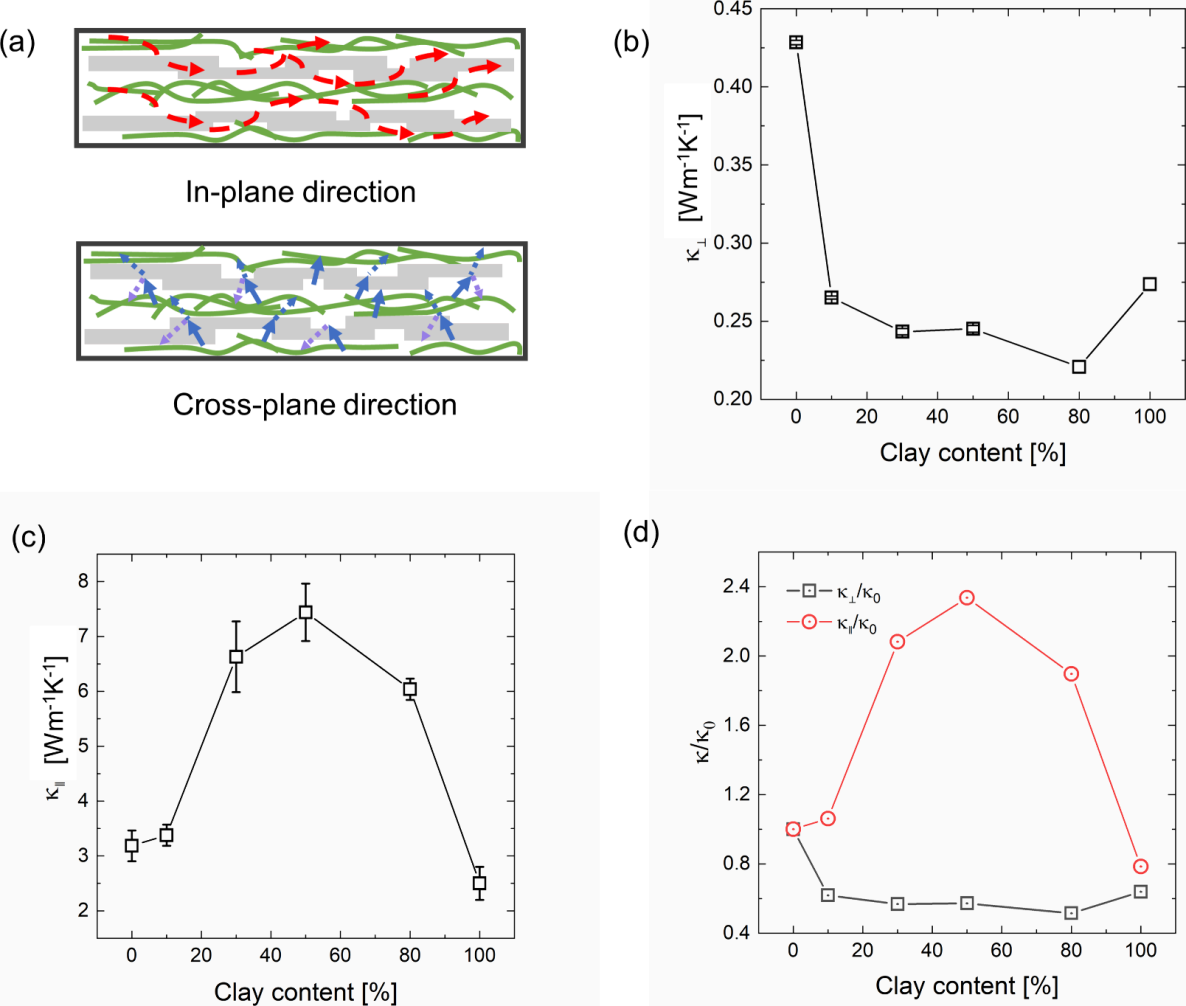
Thermal conductivity of the MTM clay/CNF hybrid composites. (a) Schematic of the in-plane and cross-plane thermal conductivity measurements. (b) Dependence of cross-plane thermal conductivity (κ_⊥_) on the clay content. (c) Dependence of in-plane thermal conductivity (κ_∥_) on the clay content. (d) Variation of the in-plane and cross-plane thermal conductivity with respect to the pure CNF case.

From the results, we found that the cross-plane thermal conductivity decreased nonmonotonically with an increase in MTM clay content (Figure 2b). The value reached a maximum of 0.42 W m^–1^ K^–1^ in the case of 0%-clay and decreased until the 80%-clay case, reaching a minimum of 0.22 W m^–1^ K^–1^. Finally, the thermal conductivity exhibited a slight increase for the pure clay case (0.27 W m^–1^ K^–1^), where the value is comparable with that of pure clay in the literature (0.1–0.2 W m^–1^ K^–1^).^19^ It is interesting to note that the cross-plane thermal conductivity of the composite was somewhat lower than that of pure clay. To validate the transient measurement of the composite via diffusivity, the cross-plane thermal conductivity was also directly measured using the steady-state method, and the obtained clay content dependence was confirmed to reasonably agree with the laser flash measurements (details in Supporting Information Figure S-4).

As shown in Figure 2, a distinct contrast was observed between the cross-plane and in-plane thermal conductivities of the clay/CNF composite, where the in-plane thermal conductivity was significantly larger than that of the cross-plane thermal conductivity. The large anisotropy is understandable considering that intrananofibril heat conduction is larger than the internanofibril heat conduction. The intrananofibril heat conduction along the fibril axis is larger because the covalent bonds within the cellulose molecule chain promotes the phonon group velocity and mean paths and the ordering of cellulose molecule chains enhances phonon transmission between the chains. On the other hand, internanofibril phonon transport is limited by weak bonding and disordered contact. Similarly, as for the montmorillonite layers, the covalent bonds in the Si–O tetrahedral framework and ordered aligned layers promote phonon transport.

Figure 2c illustrates the in-plane thermal conductivity with varying clay contents. The in-plane thermal conductivity first increased monotonically with increasing clay content and reached a high value in the case of 50% clay (7.5 W m^–1^ K^–1^), with a 150% enhancement compared to pure CNF films. With further increase in the clay content, in-plane thermal conductivity monotonically decreased to 2.5 W m^–1^ K^–1^ in the 100%-clay case, which is comparable with the reference value of 1.8–3.2 W m^–1^ K^–1^ for pure clay.^20^ The in-plane thermal conductivity varies between 2.5 Wm^–1^K^–1^ to 7.5 W m^–1^ K^–1^, and the cross-plane values are between 0.22 W m^–1^ K^–1^ and 0.43 W m^–1^ K^–1^. Figure 2d compares the enhancement ratios of the in-plane and cross-plane thermal conductivities. For in-plane thermal conductivity with 50% clay, we obtained a 220% thermal conductivity enhancement compared with the pure CNF case.^21^

Because the interaction between CNFs originates from weak forces such as van der Waals and electrostatic forces, the collective structure of the CNFs is expected to influence the directional thermal conductivity. In this study, linearly polarized Raman spectroscopy was performed to analyze the structure and gain physical insights. As described in the Experimental Section, we used the intensity of the peak at 1096 cm^–1^ originating from the C–O–C vibration along the cellulose main chain to characterize the relative number of cellulose molecules oriented in the in-plane (random) directions. The arrangements of the samples with respect to the polarization of the beam for the in-plane and cross-plane measurements are shown in Figure S-3.

Figure 3 shows a comparison of the cases with (a) pure CNF, (b) 10% clay, (c) 50% clay, and (d) 80% clay. The difference in the normalized Raman peak intensity between in-plane and cross-plane increases from the pure CNF case to the 50% clay case. This suggests that the presence of clay particles helps align CNFs in the in-plane directions, although randomly in the plane. By further increasing the clay content, the difference decreased, which may be attributed to the known increase in porosity in the composite for MTM clay contents over 50%.^15^ Notably, the porosity can also influence the degree of alignment and thermal conductivity, and the increase in the volume fraction of MTM clay contents, particularly for high clay contents, can result in high porosity as clarified in the previous study on identical composite materials.

**Figure 3:**
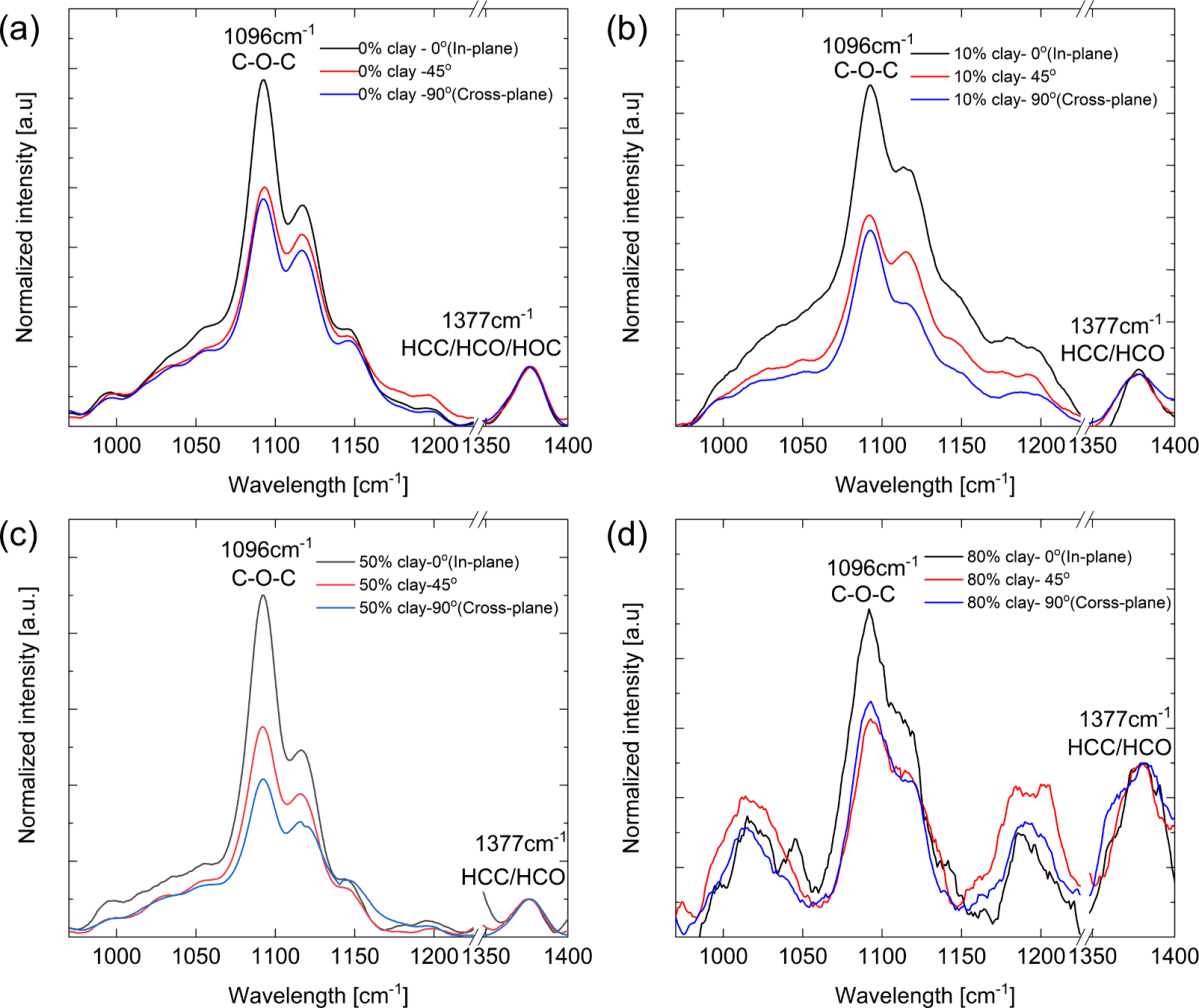
Nanocellulose structure characterization via Raman spectroscopy. A linear polarized beam was used for both incident and scattered light. Raman spectra normalized with the intensity at 1377 cm^–1^ for (a) pure-CNF, (b) 10%-clay, (c) 50%-clay, and (d) 80%-clay films.

The correlations between the normalized Raman peak intensity at 1096 cm^–1^ and thermal conductivity for cross-plane and in-plane directions are shown in Figure 4a and Figure 4b, respectively. The in-plane thermal conductivity correlated well with the Raman peak intensity, whereas the cross-plane thermal conductivity did not. The resulting thermal conductivity anisotropy is shown in Figure 4c. The good correlation between the extent of planar alignment and in-plane thermal conductivity suggests that the alignment increases the thermal conductivity. This can be explained as follows. The in-plane heat conduction through the CNF layer is dominated by the interfibril thermal conductance (or resistance). Planar alignment confines the constituent CNFs in a plane, which increases the effective area of interfibril contacts for a given number of CNFs. This increases the interfibril thermal conductance, and thus the in-plane thermal conductivity.

**Figure 4:**
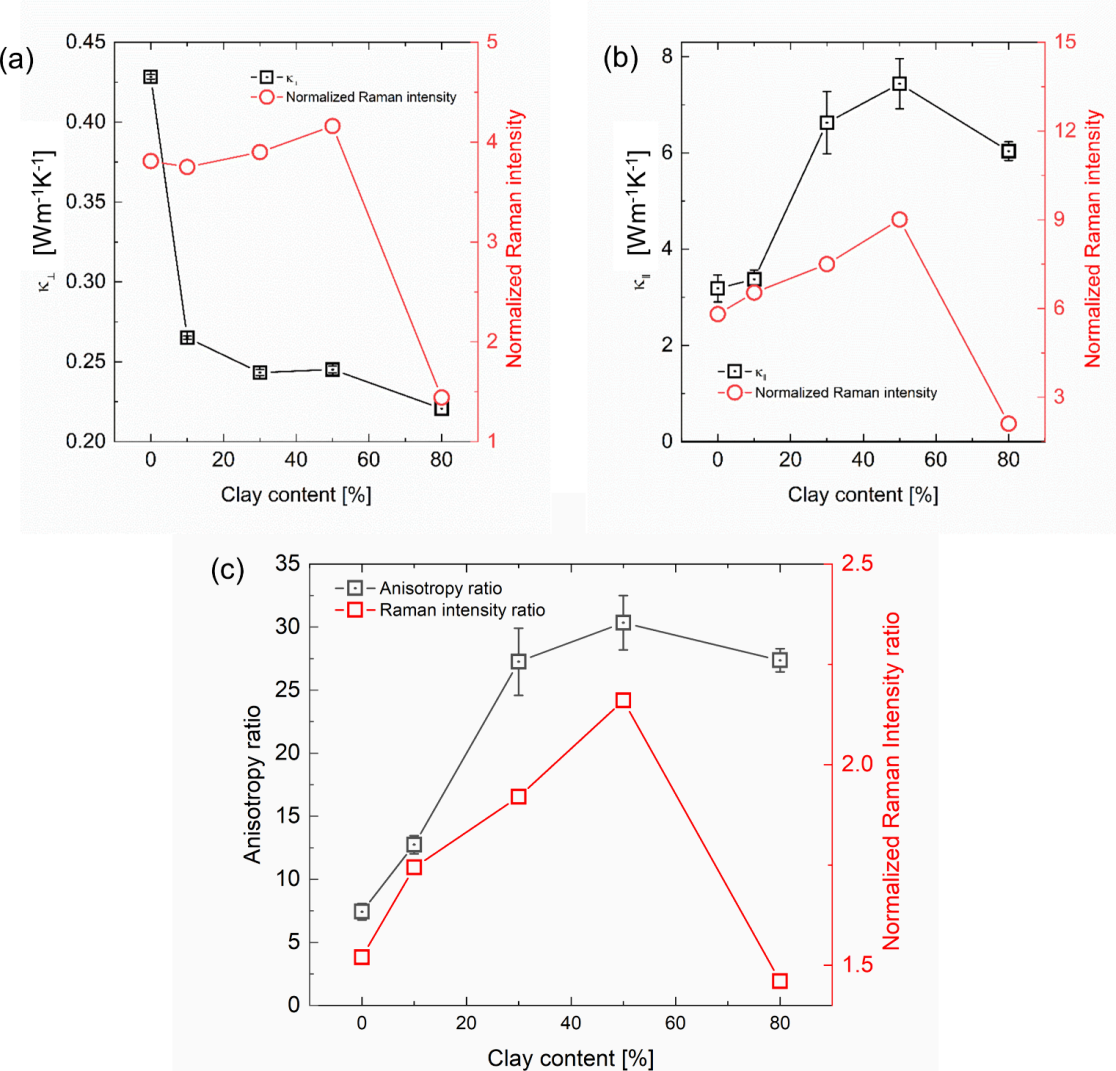
Correlations between the thermal conductivities and normalized Raman intensity at 1096 cm^–1^: (a) cross-plane thermal conductivity, (b) in-plane thermal conductivity, and (c) their anisotropy ratio.

Although the above discussion on the role of CNF alignment should be qualitatively feasible, it may not be sufficient to quantitatively explain the large enhancement in the in-plane thermal conductivity. Considering that the alignment is planar, and the CNFs are still randomly oriented in the plane, this geometrically limits the enhancement contact area. The contact between the randomly oriented rod-like CNFs is essentially a point contact, and the planar alignment enhances the number density of contacts rather than the area per contact for given number of CNFs. In this sense, the rate of enhancement of the contact area between the clay platelets should be higher, because they can contact in-plane. Because the measured degree of planar alignment of CNFs should also indicate that of the clay platelets, it should be fair to state that the thermal conductivity enhancement was caused by the alignment of both CNFs and clay platelets.

For the cross-plane direction, because the cross-plane thermal conductivity of clay is significantly smaller than that of CNF, as shown in Figure 2b, the thermal conductivity is mainly determined by the clay thermal conductivity and the interface thermal conductance (resistance) between the clay platelet CNFs. The non-negligible contribution of the interfacial thermal resistance between the clay layer and CNF layer can be seen in Figure 2b, where the cross-plane thermal conductivity of the 80% clay case is smaller than the pure clay case owing to the interfacial thermal resistance. This is in line with the work of Losego et al., where abrupt organic–clay interfaces were found to significantly enhance phonon boundary scattering and reduce interfacial thermal conductance.^22^

Finally, the anisotropy ratio of the thermal conductivity was quantified as the ratio of the in-plane thermal conductivity to the cross-plane thermal conductivity. The highest anisotropy ratio of thermal conductivity in this work is 30, a record high value compared to other dense cellulose-based thin films such as shear-oriented CNC films, tunicate nanowhiskers nanopaper, TEMPO-oxidized Sugi cellulose film, and CNF/graphene films, whose anisotropy ratios were reported to be about 2.4,^23^ 8.5, 1.8,^24^ and 23,^25^ respectively.

## Conclusion

We prepared a series of thin hybrid films composed of MTM clay and CNFs with a layer-by-layer structure using a vacuum filtration method. This composite exhibited excellent heat-shielding properties by radially dissipating localized heat at positions subjected to a flame torch or fire, while limiting the through-thickness thermal conductivity. Thermal measurements were performed in both the in-plane and through-thickness directions. The through-thickness thermal conductivity depended nonmonotonically on the clay content. As the clay content increased from 0% (pure CNFs), the through-thickness thermal conductivity decreased until the content reached 80% because of the high density of CNF/MTM interfaces and then increased at 100% (pure clay). The in-plane thermal conductivity also exhibited a nonmonotonic dependence on the clay content, with a maximum at 50% clay content. This gives rise to the largest anisotropy ratio at clay content of 50%, where in-plane thermal conductivity was 30 times greater than the through-thickness thermal conductivity. Notably, the porosity of the material increased above 50% clay content. Raman spectroscopy was performed to investigate the orientation of the CNFs in the composite, and revealed that the degree of alignment strongly depended on the clay content, with a maximum at 50% clay content. The alignment of the CNFs and clay platelets contributed to anisotropy. The high anisotropy ratio of thermal conductivity in the clay/cellulose films reported herein is an important result that may be further exploited in material design to broaden the range of applications of fire-retardant materials.

## Supplementary Material



## Notes

Thermal diffusivity measurement; heat capacity measurement; nanostructure investigation; steady-state measurement for cross-plane thermal conductivity; schematic diagram of thermal diffusivity measurement for clay/CNF composite by using the laser flash method; heat capacity measurement results for clay/CNF composite in the range of 25–140 °C by using DSC; illustration of linearly polarized Raman measurement for both incident and scattered beam to estimate orientation of CNFs from the cross-plane direction; cross-plane thermal conductivity measurement of the clay/CNF composites obtained from the steady-state method and laser flash method; in-plane thermal diffusivity measurement data by LFA (10%clay-90%CNF sample) (PDF)
