# Supplementary material for: Large Anisotropy of Thermal Conductivity in Oriented Cellulose–Clay Composites
Source: ACS Omega. 2025 Jun 16;10(25):26560–6. doi: 10.1021/acsomega.5c00316 (PMC12223830; doi:10.1021/acsomega.5c00316)
Supplement: Supplementary file 1 [file ao5c00316_si_001.pdf]

Large Anisotropy of Thermal Conductivity in Oriented Cellulose-Clay Composites

Guantong Wang[§], Lengwan Li[†], Lilian Medina[†], Sivasankaran Harish[§], Jing Liu[§], Bin Xu[§], Rulei Guo[§], Cheng Shao[§], Takashi Kodama[§], Lars A. Berglund[†,*], Junichiro Shiomi[§&*]

[§]Department of Mechanical Engineering, Graduate School of Engineering, The University of Tokyo, 7-3-1, Bunkyo-ku, Tokyo, 113-8656, Japan

[†]Department of Fiber and Polymer Technology, Wallenberg Wood Science Center, KTH Royal Institute of Technology, 10044 Stockholm, Sweden

[&]Institute of Engineering Innovation, Graduate School of Engineering, The University of Tokyo, 2-11, Yayoi, Bunkyo-ku, Tokyo, 113-0032, Japan

**Corresponding author: Junichiro Shiomi, shiomi@photon.t.u-tokyo.ac.jp; Lars A. Berglund, blund@kth.se*

ASSOCIATED CONTENT

Supplementary Information

1. Thermal diffusivity measurement.

Thermal diffusivity measurement for clay/CNF composite was done by using Laser Flash (as shown in Fig. S-1). Samples were coated with Au with 100 nm on both sides and spayed with graphite before the measurement. For the in-plane measurements, the sample was shaped to a rectangular with length and width of 25 mm and 10 mm. For the cross-plane measurements, the sample was shaped to have a square top of 8 mm × 8 mm.

Assuming a one-dimensional heat transfer inside a perfectly thermally insulated solid of uniform thickness L, and the temperature distribution inside the latter can be calculated with following equation by Carslaw and Jaeger[1]:

$$\theta(x,t) = \frac{1}{L}\int_0^L \theta(x,t)\mathrm{d}x + \frac{2}{L}\sum_{n=1}^{\infty} \exp\left(\frac{-n^2\pi^2\alpha t}{L^2}\right) \times \cos\frac{n\pi x}{L}\int_0^L \theta(x,0)\cos\frac{n\pi x}{L}\mathrm{d}x,$$

where, x is the distance from the bottom of the sample, and t is the time elapsed from laser being flashed. The adiabatic boundary conditions without any heat losses to the ambient simplifies the solution to:

$$\theta(t) = \frac{Q}{\rho C_p L}\left[1 + 2\sum_{n=1}^{\infty}(-1)^n \exp\left(\frac{-n^2\pi^2\alpha t}{L^2}\right)\right].$$

Here, Q is the energy density of laser flash pulse, ρ is density of sample, C_p is specific heat. From the solution above, Parker et al. [2] deduced a simple formula to calculate the thermal diffusivity α of the sample's material with only two experimental parameters, which are easy to evaluate.

$$\alpha = \frac{0.1388 \times L}{t_{50}},$$

where L is the thickness of the sample, and t_{50} is the half-time at which the temperature rise of on the top surface of the sample reaches half of its maximum value. The coefficient of this solution is

estimated as 0.1388.

For the cross-plane LFA model, it is based on a one-dimensional model, "Cowan +" model[3], which takes radiation and convection on both upper and bottom surfaces into account, and implemented to analyze the temperature profile. The thermal conductivity of the composite can be calculated since the volume fraction of the individual layer and hence the heat capacity and density of the whole composite were given. To extract the thermal resistance of the binder layer, a typically used multilayer heat diffusion model[4] is implemented. The key in this analysis is to obtain the real heat diffusion time from the temperature response on the rear surface of the sample, which is the accumulative area (A). A in a 3-layer model (Graphite+Sample+Graphite) is expressed as follow:

$$A = \frac{b_1\sqrt{\tau_1}\left(\frac{\tau_1}{6}+\frac{\tau_2}{2}+\frac{\tau_3}{2}\right)+b_2\sqrt{\tau_2}\left(\frac{\tau_1}{2}+\frac{\tau_2}{6}+\frac{\tau_3}{2}\right)+b_3\sqrt{\tau_3}\left(\frac{\tau_1}{2}+\frac{\tau_2}{2}+\frac{\tau_3}{6}\right)+\frac{b_1 b_3}{b_2}\sqrt{\tau_1\tau_2\tau_3}}{b_1\sqrt{\tau_1}+b_2\sqrt{\tau_2}+b_3\sqrt{\tau_3}}$$

Here, b is the thermal penetration depth and τ is the characteristic time of the heat diffusion in individual layers, the subscript 1~3 corresponds to the first, second, and third layers. The b and τ are expressed as:

$$b_i = \rho_i C_{pi}\sqrt{\alpha_i}$$

$$\tau_i = \frac{d_i^2}{\alpha_i}$$

ρ is the density, C_p is the specific heat, and α is thermal diffusivity. By substituting A obtained from the temperature decay profile to the equation, we can calculate the thermal diffusivity of the MTM/CNF layer (the second layer) and obtain the corresponding thermal resistance.

The measurement for in-plane model is a single pulse laser beam is flashed on the surface of the sample with a diameter about 10mm. The beam shape is restricted to the line-shaped area on the

sample surface with a fixed slit clamp. This gives a temperature distribution in the direction of sample width, with no temperature gradient in the cross direction (a short measuring time makes small heat leak).

The temperature dependence along x direction from the center of spot sample, as a function of time is given as:

$$\varphi(\alpha t, x) = \frac{1}{\pi} \int_{-\infty}^{\infty} \varphi_0\left(0,\ x + 2\beta\sqrt{\alpha t}\right) \exp(-\beta^2) d\beta$$

$\varphi(0, X)$ is the initial temperature distribution given as:

$$\varphi_0(\alpha t, x) = T_0\ (-l < x < L),\ 0\ \text{(others)}$$

$$T_0 = Q/\rho C_p d$$

T_0 is the temperature raise, α is thermal diffusivity, l is the half of the laser width, Q is the heat absorbed by the sample in every unit area, d is the thickness of the sample.

2. Heat capacity measurement.

From the results of the heat capacity of clay/CNF composites (as Fig. S-2), we can find that, with increasing temperature, heat capacity increases linearly. This is mainly due to the increased number of phonons in the measured temperature range. Heat capacity is the largest in pure CNF sample and decreases as the clay content increases. The heat capacity of wood cellulose increases with increasing temperature up to 160 ºC [5]. The reference heat capacity value for MTM clay (Ca-montmorillonite) and CNF (wood cellulose) are taken from the literatures [6,7]. The heating rate of DSC is 10 K/min, and it is based on the ASTM standard for C_p measurement.

The non-monotonous trend between 10%-clay and 30%-clay samples could be attributed to its high

CNF fraction, where relatively larger moisture absorption may give rise to some error, since the mass of samples were measured before putting the sample in the pans for pre-heating.

3. Nano structures investigation.

Nanocellulose orientation was investigated via linearly polarized Raman spectroscopy for both incident and scattered beam as shown in Fig. S-3. Prior to the measurement, crystalline silicon (representative peak at 520.6 cm^{-1}) is used for calibration. The measurements are done with microscope camera magnification 100X, 0.1% near-infrared laser state, and acquisition exposure time of 30/s accumulated 3 times. Measured wavenumber range for composites is from 30 to 2500 cm^{-1}. The spectral data was obtained by subtracting the baseline and smoothed.

4. Steady stead measurement for cross-plane thermal conductivity.

Steady state measurements were done to double check the cross-plane thermal conductivity of the clay/CNF samples (Fig. S-4). The sample was placed in between the two copper blocks, where the top copper block serves as hot side and the bottom block serves as cold side. The thermal contact between the copper blocks and the sample was assured by using thermal grease. Heat flux through the sample can be obtained by measuring the temperature gradient in the copper measured by thermocouples and the known thermal conductivity of copper. Then by measuring the temperature drop across the sample, the cross-plane thermal conductivity can be obtained through Fourier's law. This allows us to directly obtain the thermal conductivity without using Equation 2, whose applicability to composite materials needs to be validated.

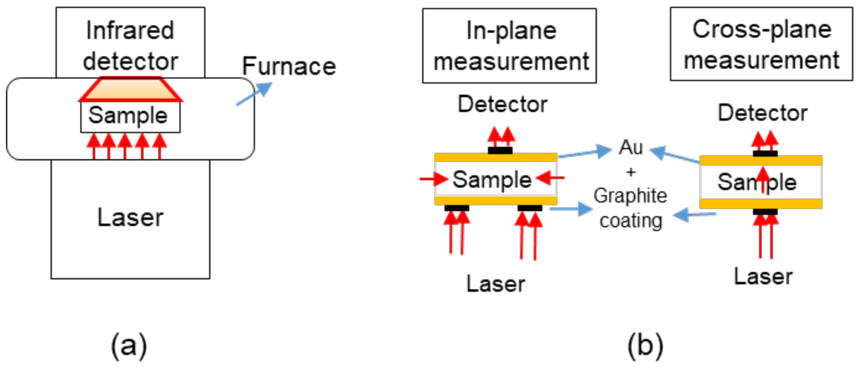

Fig. S-1 Schematic diagram of thermal diffusivity measurement for clay/CNF composite by using the laser flash method. **a** Schematic of the laser flash instrument. **b** Schematic of the in-plane and cross-plane thermal conductivity measurements, where the samples were coated with Au with 100 nm on both sides and then with graphite spray before the measurement.

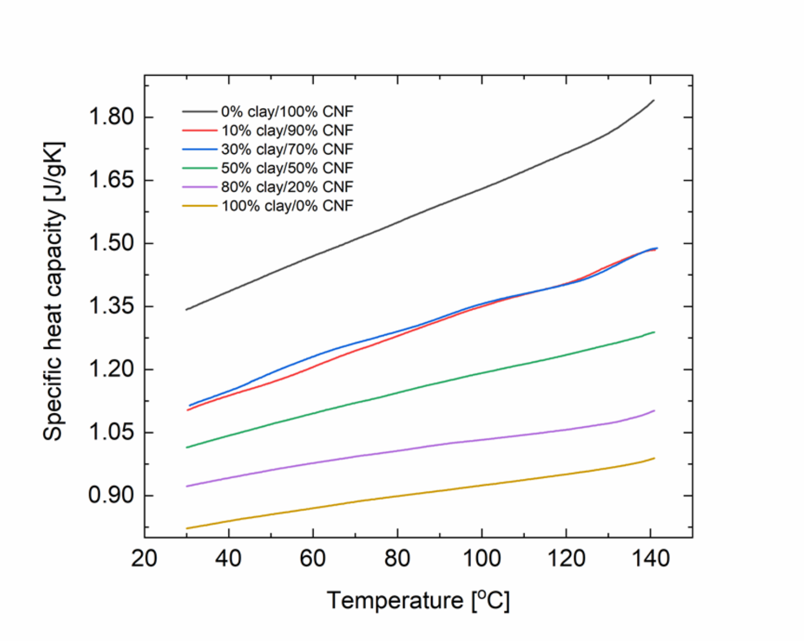

Fig. S-2 Heat capacity measurement results for clay/CNF composite in the range of 25-140℃ by using DSC. The heat capacity increases approximately linearly with increasing temperature.

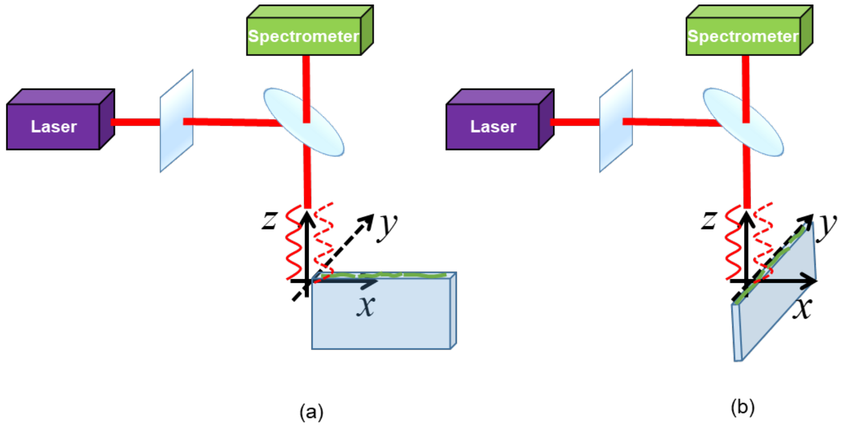

Fig. S-3 Illustration of linearly polarized Raman measurement for both incident and scattered beam to estimate orientation of CNFs from the cross-plane direction. **a** Schematic of the measurement in the in-plane direction, where the beam is polarized in the in-plane of the sample (*x*-*z* plane in the figure). **b** Schematic of the measurement in the cross-plane direction, where the beam is polarized in the cross-plane of the sample (*x*-*z* plane in the figure).

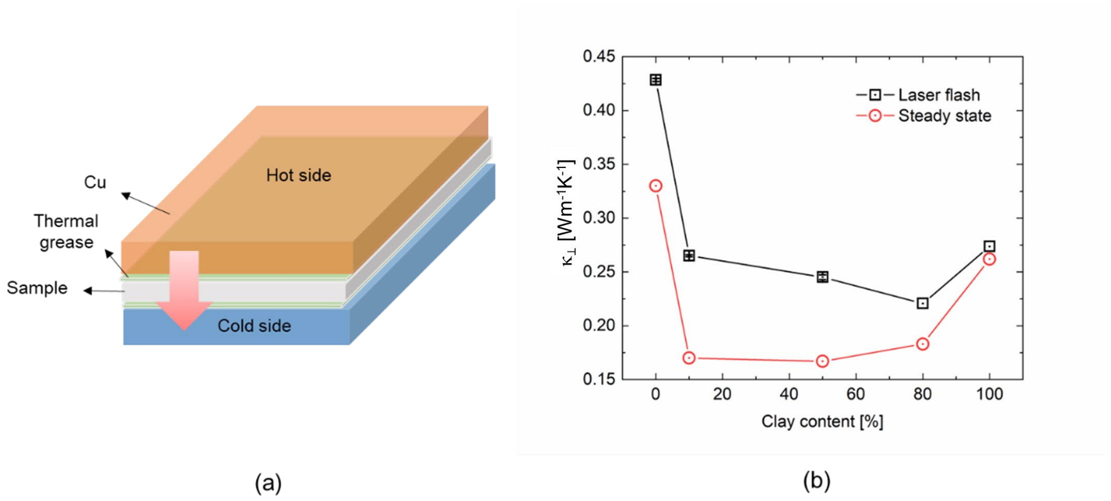

Fig. S-4 Cross-plane thermal conductivity measurement of the clay/CNF composites obtained from the steady-state method and laser flash method. **(a)** Schematic of self-built steady-state measurement setup. Temperature difference is generated from top (hot side) to bottom (cold side). (**b)** Comparison of the results of steady-state measurement and laser flash measurement.

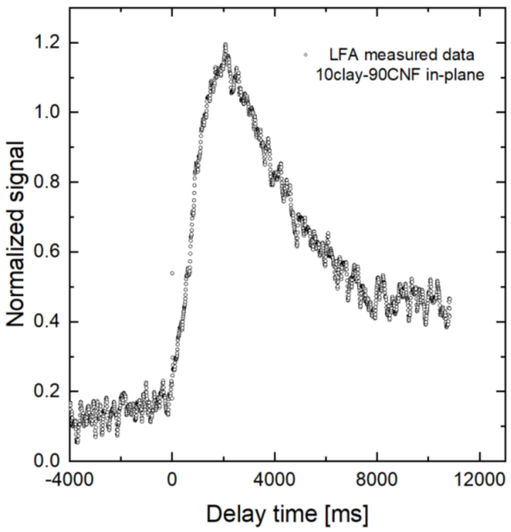

Fig. S-5 In-plane thermal diffusivity measurement data by LFA (10%clay-90 %CNF sample).

Reference:

(1) Johra, H. Description of the Laser Flash Analysis Method for Thermal Diffusivity Measurement with the LFA 447, **2019**.

(1) Johra, H. Description of the Laser Flash Analysis Method for Thermal Diffusivity Measurement with the LFA 447, URL: Https:// Vbn.Aau.Dk/ En/ Publications/ Description- of-the- Laser-Flash- Analysis- Method- for- Thermal-Diffus. **2019**.

(2) Parker, W. J.; Jenkins, R. J.; Butler, C. P.; Abbott, G. L. Flash Method of Determining Thermal Diffusivity, Heat Capacity, and Thermal Conductivity. *J. Appl. Phys.* **1961**, *32* (9), 1679–1684. https://doi.org/10.1063/1.1728417.

(3) Cowan, R. D. Pulse Method of Measuring Thermal Diffusivity at High Temperatures. *J. Appl. Phys.* **1963**, *34* (4), 926–927. https://doi.org/10.1063/1.1729564.

(4) Akoshima, M.; Tanaka, T.; Endo, S.; Tetsuya Baba; Harada, Y.; Kojima, Y.; Kawasaki, A.; Ono, F. Thermal Diffusivity Measurement for Thermal Spray Coating Attached to Substrate Using Laser Flash Method. *Jpn. J. Appl. Phys.* **2011**, *50* (11 PART 2), 0–8. https://doi.org/10.1143/JJAP.50.11RE01.

(5) Hatakeyama, T. Studies on Heat Capacity of Cellulose and Lignin by Differential Scanning Calorimetry *. **1982**, *23* (November 1801), 1801–1804.

(6) Skauge, A.; Fuller, N.; Hepler, L. G. Specific Heats of Clay Minerals: Sodium and Calcium Kaolinites, Sodium and Calcium Montmorillonites, Illite, and Attapulgite. *Thermochim. Acta* **1983**, *61* (1–2), 139–145. https://doi.org/10.1016/0040-6031(83)80310-4.

(7) Fujii, M.; Zhang, X.; Xie, H.; Ago, H.; Takahashi, K.; Ikuta, T.; Abe, H.; Shimizu, T. Measuring the Thermal Conductivity of a Single Carbon Nanotube. *Phys. Rev. Lett.* **2005**, *95* (6), 8–11. https://doi.org/10.1103/PhysRevLett.95.065502.
